# Preparation and evaluation of injectable Rasagiline mesylate dual-controlled drug delivery system for the treatment of Parkinson’s disease

**DOI:** 10.1080/10717544.2017.1419514

**Published:** 2017-12-23

**Authors:** Ying Jiang, Xuemei Zhang, Hongjie Mu, Hongchen Hua, Dongyu Duan, Xiuju Yan, Yiyun Wang, Qingqing Meng, Xiaoyan Lu, Aiping Wang, Wanhui Liu, Youxin Li, Kaoxiang Sun

**Affiliations:** ^a^ School of Pharmacy, Collaborative Innovation Center of Advanced Drug Delivery System and Biotech Drugs in Universities of Shandong, Key Laboratory of Molecular Pharmacology and Drug Evaluation (Yantai University), Ministry of Education, Yantai University Yantai Shandong Province PR China; ^b^ State Key Laboratory of Long-Acting and Targeting Drug Delivery System, Shandong Luye Pharmaceutical Co., Ltd Yantai Shandong Province PR China

**Keywords:** Sustained release, *in situ* forming implant, microspheres, water-soluble drug, MAO-B inhibitor

## Abstract

A microsphere–gel *in situ* forming implant (MS–Gel ISFI) dual-controlled drug delivery system was applied to a high water-soluble small-molecule compound Rasagiline mesylate (RM) for effective treatment of Parkinson’s disease. This injectable complex depot system combined an *in situ* phase transition gel with high drug-loading and encapsulation efficiency RM–MS prepared by a modified emulsion-phase separation method and optimized by Box–Behnken design. It was evaluated for *in vitro* drug release, *in vivo* pharmacokinetics, and *in vivo* pharmacodynamics. We found that the RM-MS-Gel ISFI system showed no initial burst release and had a long period of *in vitro* drug release (60 days). An *in vivo* pharmacokinetic study indicated a significant reduction (*p* < .01) in the initial high plasma drug concentration of the RM–MS–Gel ISFI system compared to that of the single RM–MS and RM–*in situ* gel systems after intramuscular injection to rats. A pharmacodynamic study demonstrated a significant reduction (*p* < .05) in 6-hydroxydopamine-induced contralateral rotation behavior and an effective improvement (*p* < .05) in dopamine levels in the striatum of the lesioned side after 28 days in animals treated with the RM–MS–Gel ISFI compared with that of animals treated with saline. MS-embedded *in situ* phase transition gel is superior for use as a biodegradable and injectable sustained drug delivery system with a low initial burst and long period of drug release for highly hydrophilic small molecule drugs.

## Introduction

Parkinson’s disease (PD) is a common progressive neurodegeneration disorder, second only to Alzheimer’s disease (AD). PD affected 6.2 million people globally and led to approximately 117,400 deaths in 2015 (Collaborators, 2016a,b). Approximately one percent of the population over age 60 suffer from the disease (Ellis & Fell, 2017). Although PD is incurable, symptomatic therapies can improve the quality of life of patients (Connolly & Lang, 2014). Monoamine oxidase (MAO)-B inhibitors exert a moderate benefit on PD motor symptoms, fatigue, and mood when used in early PD, and also play a marginal role as an adjunct therapy with levodopa in advanced PD (Marsili et al., 2017).

Rasagiline mesylate (RM, Azilect^®^) is a second generation, highly potent selective and irreversible MAO-B inhibitor that reduces the catabolism of dopamine (DA), and, therefore, increases the availability of neurotransmitters at the synaptic level (Garbayo et al., 2013; Weinreb et al., 2015). However, the clinical applications of RM are limited by its low oral bioavailability of only 36% and its requirement of daily administrations, since it has a short elimination half-life (Dashtipour, 2007). In addition, Yoram et al. (2012) confirmed that RM administered in a sustained release manner led to an increase in DA levels that were much higher than those from the same dose of an acute oral formulation in 1-methyl-4-phenyl-1,2,3,6-tetrahydropyridine (MPTP)-treated mouse brains. Therefore, RM is a suitable candidate for formulation in a controlled drug delivery system.

Encapsulating the active ingredients within a biodegradable polymeric microsphere is a good choice to achieve the desired controlled release effect because the polymer acts as a rate-controlling membrane after injected into the body (Ng et al., 2010). Fernandez et al. (2012) encapsulated RM into poly (lactic-co-glycolic acid) (PLGA) microspheres (MS) by emulsion-solvent evaporation methods (oil-in-water (O/W) emulsion and water-in-oil-in-water (W/O/W) double emulsion). However, according to the results, RM–MS with a low loading content (3–5 mg per 100 mg PLGA) and a clear burst release (10–25% for 1 h) showed a constant *in vitro* drug release for only two weeks. Obviously, the currently available RM microsphere agents cannot satisfy the needs of daily clinical treatment.

*In situ* forming implant (ISFI) systems have received widespread interest over recent decades attributed to a range of advantages, including systemic and prolonged drug delivery with low side effects, easy and less invasive application, protection of drugs, biodegradability and biocompatibility (Juvekar & Kathpalia, 2017). Importantly, ISFI systems are compatible with various materials, ranging from drug molecules (Wang et al., 2012; Avachat & Kapure, 2014; Karfeld-Sulzer et al., 2015) to drug-loaded particles, such as nanoparticles (Bisht et al., 2017) and microparticles (Bege et al., 2013; Wang et al., 2017).

Lin et al. (2015) developed a uniform ultra-small microsphere/sucrose acetate isobutyrate (SAIB) hybrid depot to provide a long-term sustained release system for the water-insoluble drug, Risperidone; it not only reduced the burst release of the SAIB depot, but also eliminated the lag-time of the PLGA microspheres. It is worth considering that most existing clinical medications are hydrophilic small-molecule compounds, and compared to that of water-insoluble drugs, the quick and unstable drug release profiles make it tougher for controlled release of water-soluble drugs. Therefore, it is very useful to apply this hybrid depot system clinically to controlled-release formulations of water-soluble small-molecule drugs.

In this study, we first compared the effect of encapsulating the highly water-soluble small-molecule compound, RM, into PLGA MS using emulsion-solvent evaporation methods and phase separation methods, and then selected an appropriate preparation method for optimization by design of experiments (DOE) to obtain high encapsulation efficiency. Subsequently, we applied the MS–Gel ISFI dual-controlled drug delivery system to overcome the weaknesses of the unsatisfactory drug release pattern of both RM–*in situ* gel and RM-MS systems. In this study, RM–MS, RM–*in situ* gel, and RM–MS-Gel ISFI were prepared and evaluated by *in vitro* drug release and *in vivo* pharmacokinetic studies. In addition, a pharmacodynamic study was performed to evaluate the treatment effects of RM–MS–Gel ISFI in a rat model of 6-hydroxydopamine (6-OHDA)-induced PD.

## Experimental section

### Materials

SAIB, Mw 856, specific gravity 1.146 kg L^−1^ at 25 °C, was obtained from Eastman Co., Ltd., St. Louis, MO, USA. Lakeshore^®^ 7525 DLG 2 A (poly-d,l-lactide-co-glycolide, 75:25), Mw 14,000 Da, inherent viscosity 0.17 dL g^−1^ was purchased from SurModics Pharmaceuticals, Inc., Birmingham, AL, USA. RM (98%) was bought from Yancheng Langde Chemical Co., Ltd., Yancheng, China. Dimethicone, viscosity 350 cSt, was obtained from Dow Corning Corporation, Midland, MI, USA. Ethanol (EtOH), *n*-methylpyrrolidone (NMP), dichloromethane (CH_2_Cl_2_), and n-heptane were purchased from Sinopharm Chemical Reagent Co., Ltd., Shanghai, China. Span 80 was obtained from Nanjing Well Chemical Co., Ltd., Nanjing, China. Water was purified with a Milli-Q^®^ (Bedford, MA, USA) filtration system, Millipore, USA. All other reagents and solvents were analytical grade.

### Preparation of RM–MS

The RM–MS prepared by emulsion-solvent evaporation consisted of three methods: O/W single emulsion (method A) (Fernandez et al., 2011), W/O/W double emulsion (method B) (Fernandez et al., 2009), and solid-in-oil-in-water (S/O/W) double emulsion (method C) (Paillard-Giteau et al., 2010). Briefly, PLGA was dissolved in CH_2_Cl_2_ to prepare the organic phase. RM was dissolved or dispersed into suitable additives and was then mixed with the organic phase. The mixtures of methods B and C were emulsified by using the Ultra-Turrax^®^ system (IKA T25, Staufen, Germany) with ice-cooling. The resulting O solution, W/O, and S/O primary emulsions were emulsified into the external phase consisting of PVA solution to remove the organic solvent.

The RM–MS prepared by phase separation consisted of two methods: O/O traditional phase separation (method D) and W/O/O emulsion-phase separation (method E) (David et al., 1997). In short, PLGA was dissolved in CH_2_Cl_2_ followed by the addition of RM dissolved in appropriate solvents. The mixture of method E was intensively emulsified by means of an Ultrasonic Cell Crusher (XinZhi JY92-II, Ningbo, China) at 400 W with ice-cooling. Phase separation was achieved by adding dimethicone. The resulting soft-spheres were gently transferred to an external phase of heptane and Span-80 mixed solution and stirred with ice-cooling for solidification.

### Optimization of RM-MS

The DOE technique was used to optimize the preparation process for RM–MS. Preliminary results indicated that the variables mostly affecting the quality of MS prepared by method E were the amount of the drug, the volume ratio of organic (CH_2_Cl_2_) to aqueous (water) phase of the primary emulsion, and the stirring speed during solidification. A three-factor, three-level factorial Box–Behnken design (Table S1) was used to statistically optimize the formulation parameter variables (Cavazzuti, 2013). Design-Expert^®^ software (v.8.0.6 Stat-Ease, Minneapolis, MN, USA) was used for the creation and evaluation of the experimental design.

### Preparation of RM–in situ gel

The gel matrix solutions were prepared by dissolving SAIB in appropriate amounts of organic solvents (Table 1) under ultrasonic vibration for 1 h in a water bath at 50 °C. Thirty milligrams of RM was dissolved in 1 mL SAIB matrix solution using ultrasonic vibration for 30 min. The final drug content of RM–*in situ* gel was 30 mg/mL.

**Table 1.** Formulations of RM–*in situ* gel and RM–MS–Gel ISFI systems.

Batches	RM–MS (mg)	RM (mg)	SAIB (mg)	EtOH (mL)	NMP (mL)
S1	100	–	850	0.15	–
S2	100	–	800	0.20	–
S3	100	–	750	0.25	–
S4	100	–	750	–	0.25
S5	100	–	700	–	0.30
S6	100	–	600	–	0.40
S7	–	30	850	0.15	
S8	–	30	750	–	0.25

### Preparation of RM–MS–gel ISFI

Prior to the *in vitro* drug release, *in vivo* pharmacokinetics and *in vivo* pharmacodynamics, 100 mg RM-MS (containing 30 mg RM) was suspended in 1 mL SAIB matrix solution (Table 1), with vortexing for 1 min to form the RM–MS–Gel ISFI. The final drug content of RM–MS–Gel ISFI was 30 mg/mL.

### Characterization

#### Particle size and distribution

Particle size and size distribution were measured using a laser diffraction particle size analyzer (Mastersizer 2000, Malvern, UK). The RM–MS were suspended in 0.1% Tween^®^ 20 solution with a stirring speed of 2200 rpm to prevent clumping. The monodispersity of the MS particles was described using the particle size dispersal coefficient (span), where a larger span value of MS represents a wider range of diameters.

#### Surface morphology

The surface morphology of the RM–MS was examined with a cold-cathode field-emission scanning electron microscope (FE-SEM; HITACHI S-4800, Japan). The samples were mounted onto double-sided adhesive tape on the sample stage and were gold sputter-coated. The acceleration voltage of the FE-SEM was 15 kV.

#### Drug loading and encapsulation efficiency

RM drug loading (DL) and encapsulation efficiency (EE) of RM–MS were investigated by the following steps. Ten milligrams of RM–MS was dissolved in acetonitrile, ultrasonically disrupted for 30 min, and then brought to 10 mL with acetonitrile. The RM solution was filtered through 0.45-μm filters and quantified by an HPLC method, detecting the material by its absorbance at 265 nm (Ravi et al., 2015). DL and EE were calculated by the following formulae:

(1) DL (%)= (weight of drug in MS/weight of MS)×100%

(2) EE (%)=(weight of drug in MS/weight of drug used in formulation) ×100%

#### Viscosity measurements

The viscosity measurements of gel matrix solutions were examined using a Digital Rotation Viscometer (BROOKFIELD; DV-C, USA) at 25 ± 0.1 °C. The relative viscosity was recorded when the torque values were between 50% and 80% by changing the rotor speed from 50 to 10 rpm.

### In vitro drug release study

A known amount of RM-MS was suspended in 3 mL phosphate-buffered saline (PBS, pH 7.4) in an EP tube. RM–*in situ* gel or RM–MS–Gel ISFI containing the same dose of RM was injected into the bottom of an EP tube with 3 mL PBS solution (pH 7.4). All the tubes were placed in a water bath shaker (SHKE7000-1CE; Thermo Scientific™, Marietta, OH, USA) at 37 °C with constant agitation of 50 rpm. EP tubes containing the RM-MS were centrifuged at 2028*g* for 15 min before sampling and then vortexed to re-suspension after sampling. At 0.04, 0.125, 0.25, 1, 2, 4, 6, 8, 10, 12, 16, 20, 24, 28, 36, 48, and 60 days, 2.5 mL of supernatant was sampled and replaced with the same volume of fresh medium. The supernatant was filtered through a 0.45-μm filter and assayed by HPLC (Ravi et al., 2015). Data were analyzed by fitting to zero-order, first-order, Higuchi, and Korsmeyer–Peppas kinetic models. The most appropriate model was selected based on the best goodness of fit.

### Pharmacokinetic study

#### Animal procedure

Male Sprague–Dawley rats (Beijing Vital River Laboratory Animal Technology Co., Ltd., Beijing, China, 200–220 g) were fed in a clean vivarium at 22 ± 2 °C on a 12 h light-dark cycle and with water and food ad libitum. All animal experimentations were approved by the St. Louis VA Animal Care Committee and conducted according to the Guiding Principles for Research Involving Animals and Human Beings.

Rats were divided into three groups (*n* = 5 per group): RM–MS aqueous suspension, RM–*in situ* gel, and RM–MS–Gel ISFI groups. Formulations were injected intramuscularly into the right hind leg muscle of the rats at a single dose of 5.6 mg/kg. Blood samples of 0.5 mL were collected at 0.04, 0.125, 0.25, 1, 2, 4, 6, 8, 10, 12, 14, 16, 18, 20, 24, 28, and 32 days post-injection via retro-orbital puncture.

#### Sample analysis

Plasma concentrations were measured by high-performance liquid chromatography–tandem mass spectrometry (HPLC–MS/MS; AB Sciex API 4500 triple-quadrupole mass spectrometer, USA; SHIMADZU LC-30 A high performance liquid chromatograph, Japan). The separation of RM was achieved using a ZORBAX SB-C_18_ column (100 × 2.1 mm, 5 μm, Agilent, USA) with a mobile phase consisting of acetonitrile: 0.1% formic acid (50:50, v/v). The flow rate was 1.0 mL·min^−1^. The mass spectrometer was operated using electrospray ionization (ESI) source with positive ion detection in multiple reaction monitoring (MRM) mode. The ion spray voltage was 5500 V, source temperature was 500 °C, declustering potential (DP) was 33 V, collision energy (CE) was 20 eV, and ion transitions were *m/z* 172.1→*m/z* 117.0 for RM and *m/z* 175.1→*m/z* 117.0 for the ^13^C_3_-RM internal standard with a dwell time of 100 ms (Ma et al., 2008; Wang et al., 2016). Data were processed with statistics software DAS v.2.0 (Mathematical Pharmacology Professional Committee of China, China), using a one-compartment model.

### Pharmacodynamic study

#### Pathological lesion model

Male Sprague–Dawley rats (200–220 g) were deeply anesthetized with 10% chloral hydrate solution (35 mg/kg) and then immobilized on a NEUROSTAR robot stereotaxic apparatus (Stereo Drive, Germany). 6-OHDA saline solution (4 mg/mL) containing 0.1% ascorbic acid was injected into the left striatum using a syringe pump (KD Scientific, USA). Twenty-one days after the surgery, the number of lateral rotations of the rats was recorded after apomorphine injection. Rats with >75 rotations per 15 min were considered to be successful models (Afshin-Majd et al., 2017).

#### Treatments and behavioral testing

Rats with left striatum lesions were divided into five groups (*n* = 5 per group): control, RM solution, and low-, medium-, and high-dose RM–MS–Gel ISFI groups. Saline solution and RM in saline were administered intramuscularly (0.5 mg/kg/day) to the control group and RM solution group, respectively, for 28 days. RM-MS-Gel ISFIs (equivalent to 3, 15, and 30 mg/kg RM) were administered intramuscularly to the low-, medium-, and high-dose groups, respectively, as a single dose. Lateral rotation tests were performed on days 0, 7, 14, 21, and 28 after administration. The numbers of rotations in 15 min was recorded.

#### Determination of striatal DA

Rats were euthanized after sedation at the end of the behavioral testing (on day 28 after administration). The striata were quickly removed from the brain on ice, then weighted and prepared for HPLC–MS/MS detection to measurement the level of DA. Data were expressed as nanogram per gram wet tissue.

### Statistical analysis

All the experiments were repeated at least three times. The data are expressed as mean ± standard deviations (SD). Dependent *t*-tests and one-way ANOVA were used to analyze the data via SPSS software. *p* < .05 were considered significant.

## Results and discussion

### Preparation of RM–MS

Two different microencapsulation processes, including emulsion–solvent evaporation and phase separation techniques by five different methods (A–E), were used to entrap the RM (water solubility >100 mg/mL) into PLGA MS to achieve a high DL, EE, appropriate particle size and distribution. The mean evaluation indicators of the formulation processing are summarized in Table S2. According to the results, the DL of RM–MS increased greatly from 1.01% to 26.42% by adopting the emulsion–phase separation technique (method E). Meanwhile, the EE of RM–MS remarkably increased as the W/O primary emulsion formed in method B (78.22%) and E (88.07%). The particle sizes and span values produced by all methods were in acceptable ranges for MS for intramuscular injection (i.e. particle sizes from 10 to 100 μm, span values of approximately 1).

In method A, the DL and EE using an O/W single emulsion were low because of a very high concentration of RM in the external water phase. RM tended to disperse in the PVA aqueous solution, rather than in the PLGA/CH_2_Cl_2_/methanol organic solution when the polymers encapsulated the drug to create the MS.

In methods B and C, DL and EE were improved because the W/O or S/O primary emulsions prepared through homogenization effectively prevented the RM from contacting the external water phase, so that it could remain in the emulsions during the formation of the MS. However, the decreased EE that occurred with the S/O/W double emulsion method implies that the W/O primary emulsion stabilizes the RM better than the S/O primary emulsion does.

Changing the external PVA aqueous solution of the W/O/W double emulsion to the heptane organic solution of the W/O/O double emulsion (method E) increases both the DL and EE by preventing RM from escaping into the external phase. The RM internal aqueous phase was strongly inclined to disperse into the W/O primary emulsion to be effectively entrapped by the PLGA via precipitation from the CH_2_Cl_2_/dimethicone hybrid solvent. Furthermore, EE increased from 79.12% to 88.07% when the concentration of RM increased from 9% to 30%, perhaps due to the decreased loss of RM during the transition process.

For the O/O phase separation method (method D), the hydrophilic RM had affinity for neither the hydrophobic PLGA/CH_2_Cl_2_ solution nor the heptane solution, which led to a lower DL and EE compared to that of the W/O/O emulsion–phase separation method.

**Figure 1.**
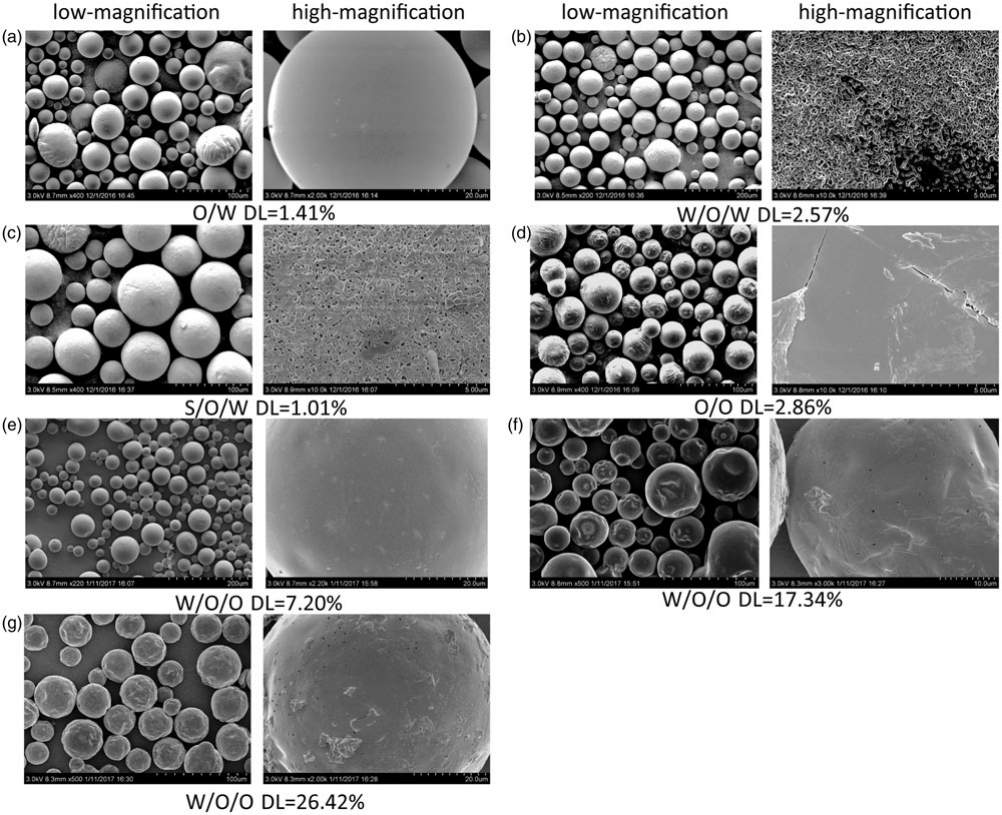
Low-magnification and high-magnification cold-cathode field-emission scanning electron microscope (FE-SEM) microphotographs of RM-microspheres prepared by (a) O/W single emulsion-solvent evaporation method, (b) W/O/W double emulsion-solvent evaporation method, (c) S/O/W double emulsion–solvent evaporation method, (d) O/O traditional phase separation method and W/O/O emulsion–phase separation method with the drug loading of (e) 7.20%, (f) 17.34% and (g) 26.42%, respectively.

FE-SEM was used to investigate the effect of the preparation methods on the particle surfaces; the photographs are shown in Figure 1. The surfaces of the RM–MS of methods A (Figure 1(a)) and D (Figure 1(d)) observed by FE-SEM were spherical and smooth, with an absence of drug crystals. Small porous channels were found on the surfaces of the RM–MS formed with the double emulsion methods B (Figure 1(b)), C (Figure 1(c)), and E (Figure 1(e–g)), which were caused by the escaping of the internal aqueous phase to the external aqueous phase (0.25% PVA solution) or external organic phase (heptane) during the solidification of PLGA. The existence of the external aqueous phase of method B resulted in a fast water release, causing an irregular, porous surface. In contrast, the MS surface prepared by method E (Figure 1(e)), with an external organic phase, was uniform, round, and less porous, which was caused by a slow water release rate. Slight invaginations and corrugations were observed on the MS surface (Figure 1(f,g)) when using method E with a high concentration of drug (DL >10%). This is probably because of the RM located close to or embedded in the particle surface during the escaping of the inner water phase.

### Optimization of RM–MS

Based on the results above, we conclude that the PLGA MS containing RM can be successfully prepared by using an emulsion–phase separation technique (method E), which achieves a high DL and EE, an appropriate particle size and distribution, and a spherical and porous surface.

A Box–Behnken experimental design, followed by response surface methodology, was applied to obtain an optimized formulation of method E for subsequent studies. Independent variables and responses are shown in Table S3. The quadratic polynomial regression fitting analysis by the Design Expert software suggested the following simplified models for the responses, *Y*_1_, *Y*_2_, and *Y*_3_, after deletion of the nonsignificant terms (*p* > .05):

(3) $$Y_{\text{1}}=\text{ 29}.\text{11}-\text{6}.\text{57}X_{\text{1}}+\text{1}.0\text{6}X_{\text{2}}+\text{1}.\text{2}0\;X_{\text{1}}X_{\text{2}}-\text{1}.\text{76 }X_{\text{2}}X_{\text{3}}-\text{9}.\text{96}X_{\text{1}}{}^{\text{2}}-\text{2}.\text{22}X_{\text{2}}{}^{\text{2}}$$

(4) $$Y_{\text{2}}=\text{ 82}.\text{93}-\text{26}.\text{7}0X_{\text{1}}+\text{2}.\text{72}X_{\text{2}}+\text{3}.\text{21 }X_{\text{1}}X_{\text{2}}-\text{5}.\text{18 }X_{\text{2}}X_{\text{3}}-\text{24}.\text{4}0X_{\text{1}}{}^{\text{2}}-\text{6}.\text{29}X_{\text{2}}{}^{\text{2}}$$

(5) $$Y_{\text{3}}=\text{ 69}.\text{4}0+\text{7}.\text{67}X_{\text{1}}-\text{1}0.\text{72}X_{\text{3}}+\text{9}.\text{46}X_{\text{1}}{}^{\text{2}}+\text{12}.\text{54}X_{\text{2}}{}^{\text{2}}+\text{12}.\text{1}0X_{\text{3}}{}^{\text{2}}$$

The main factors, *X*_1_, *X*_2_, and *X*_3_, represent the response results of changing one variable at a time from the range of low level to high level, while the interaction terms, *X*_1_ *X*_2_, *X*_2_ *X*_3_, *X*_1_^2^, *X*_2_^2^, and *X*_3_^2^, represent the response results of changing two variables at the same time. The values of the coefficients of determination (*R* ^2^) of the equations above were found to be 0.988, 0.991, and 0.913, respectively, indicating good fit.

**Figure 2.**
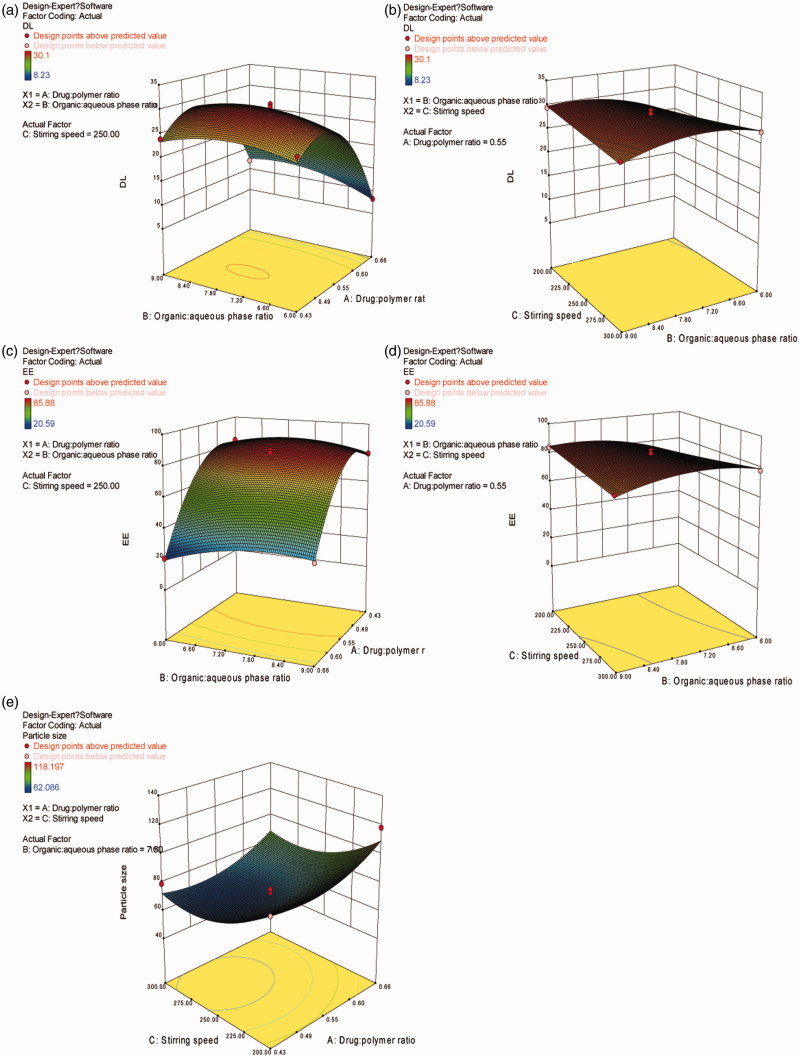
Response surface plots illustrating the effect of ratio of drug to polymer and ratio of organic to aqueous phase on (a) drug loading and (c) encapsulation efficiency (fixed stirring speed = 250 rpm), ratio of organic to aqueous phase and stirring speed on (b) drug loading and (d) encapsulation efficiency (fixed ratio of drug to polymer =1:1.8), ratio of drug to polymer and stirring speed on (e) particle size (fixed ratio of organic to aqueous phase = 7.5:1).

From Eqs. (3) and (4), *X*_1_ (ratio of drug to polymer) and *X*_2_ (ratio of organic to aqueous phase) had highly significant effects on both the DL and EE of RM–MS. However, *X*_1_ had a slightly greater contribution than did *X*_2_. The interaction of *X*_1_ *X*_2_ and *X*_2_ *X*_3_ indicated significant effects of the corresponding factors on the responses, *Y*_1_ and *Y*_2_. The response surface and contour plots of the effect of interaction of *X*_1_ *X*_2_ at a middle level of *X*_3_ (stirring speed =250 rpm) on *Y*_1_ and *Y*_2_ are shown in Figures 2(a,c). As the ratio of drug to polymer (1:2.3 to 1:1.8) and organic to aqueous phase (6:1 to 7.5:1) increased, both DL and EE increased. However, decreased DL and EE were observed when increasing the ratio of drug to polymer from 1:1.8 to 1:1.5 and the organic to aqueous phase from 7.5:1 to 9:1. This is probably due to limitations in the loading capacity of PLGA for RM, and the lower emulsification effectiveness of the primary emulsion caused by the increased volume proportion of the water phase.

The interactions of *X*_2_ *X*_3_ at a middle level of *X*_1_ (ratio of drug to polymer =1:1.8) on responses, *Y*_1_ and *Y*_2_, are demonstrated in Figures 2(b,d). Comparing Figures 2(a–d), it can be concluded that *X*_1_ *X*_2_, with a sharp response surface, has a more remarkable interaction than does *X*_2_ *X*_3_, with a gentle response surface.

It can be observed from Eq. (5) that *X*_1_ (ratio of drug to polymer) and *X*_3_ (stirring speed) had a strong effect on the particle size of RM–MS. The final particle size was mainly dependent on the shear stresses of the stirring paddle during solidification of the RM–MS. Higher stirring speeds result in stronger shear stress, producing a smaller particle size (Figure 2(e)). Moreover, when the ratio of drug to polymer increases, the amount of drug also increases; this leads to larger particle sizes.

A computerized optimization was performed by response surface analysis to obtain the optimum values of the independent variables. The optimized responses were: a ratio of drug to polymer of 1:2, a ratio of organic to aqueous phase of 7.46:1, and a stirring speed of 272 rpm. The predicted response values were: DL = 30.12%, EE = 89.88%, and particle size = 63.681 μm.

### In vitro drug release

**Figure 3.**
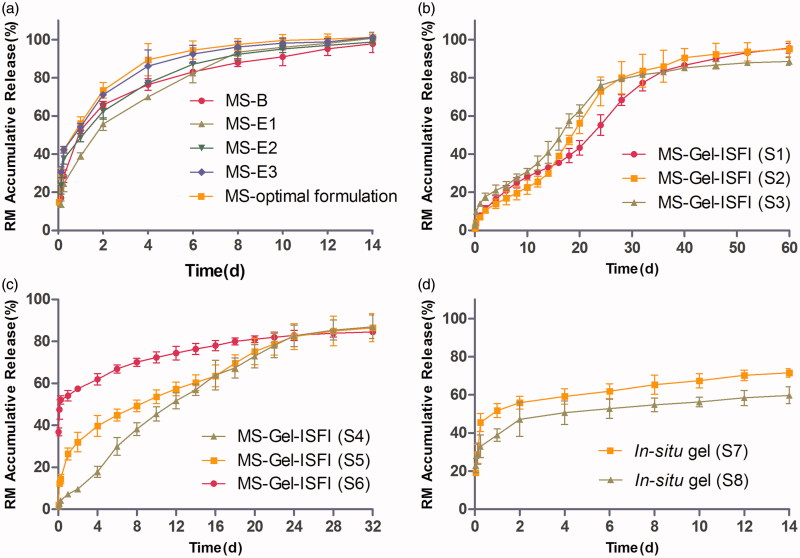
(a) The *in vitro* release profile of RM from microspheres prepared by W/O/W double emulsion–solvent evaporation method (MS-B) and W/O/O emulsion-phase separation method with the drug loading of 7.20% (MS-E1), 17.34% (MS-E2), 26.42% (MS-E3) and 30.12% (MS-optimal formulation). (b) and (c) The effect of solvent type and volume in gel matrix solution on the *in vitro* release of RM-microsphere-Gel *in situ* forming implant, 15% EtOH (S1), 20% EtOH (S2), 25% EtOH (S3), 25% NMP (S4), 30% NMP (S5), 40% NMP (S6). (d) The *in vitro* release profile of RM from *in situ* gel with the solvent of 15% EtOH (S7) and 25% NMP (S8). Graphs symbolize mean ± SD. (*n* = 3).

The release behavior profiles of RM from the PLGA MS obtained by five formulations (B, E1, E2, E3, and the optimal formulation) are shown in Figure 3(a). These profiles display a biphasic pattern, consisting of an initial burst in the first 24 h (51%, 38%, 48%, 53%, and 55%, respectively), followed by a slower sustained release in the next 14 days to release almost 100% of the RM.

For the RM–*in situ* gel system, consisting of formulations S7 and S8, the burst release (52% and 39%, respectively) was still high (Figure 3(d)). Upon injection into the medium, this system came in contact with aqueous medium, phase inversion solvent (NMP or EtOH) diffused into the aqueous medium, and water from the medium diffused into the gel matrix solution. Finally, water-insoluble gel matrix precipitated in the medium and formed a semisolid implant (Avachat & Kapure, 2014). However, during the lag time between injection into the medium and formation of the semisolid implant, a relatively large amount of drug escaped from the surface of the *in situ* gel system to the aqueous medium by means of solvent exchange, which indicates that the *in situ* gel system would not be useful to overcome the problem of burst release. The RM from the *in situ* gel was released at a very slow rate during the following 14 days, and the cumulative release of RM was only 60–70%, possibly due to delayed degradation of SAIB in pH 7.4 medium.

The RM release curve of the RM–MS–Gel ISFI system, consisting of formulations S1-S6 (Figure 3(b,c)), was completely different from that of the RM–MS and RM–*in situ* gel systems. No obvious burst release was observed in formulations S1, S2, and S4 (cumulative release of RM was only 5% in the first 24 h), indicating that drug escape was effectively prevented by the dual-controlled system combining *in situ* gel with RM-MS. During the formation of the semisolid implant, drug release was strongly inhibited by the controlled release effect of the PLGA MS, which contributed to a low drug diffusion rate from MS into the *in-situ* gel. Only a small amount of drug derived from the MS surface would be released into the medium during this lag time (Lin et al., 2015). Formulations S3 and S5 had a low burst release (13% and 24%, respectively), while formulation S6 exhibited a high initial burst (48%). This demonstrated that the controlled release effect of the drug by the MS–Gel ISFI system can be decreased by increasing the portion of solvent used in gel matrix solution preparation. RM was constantly released from the MS–Gel ISFI system, and was completely dissolved (cumulative release >85%) after 60 days with EtOH solvent (formulations S1–S3) and after 32 days with NMP solvent (formulations S4–S6).

Viscosity measurements of different concentrations of gel matrix solutions were conducted to further explain the *in vitro* release behavior of the RM from the MS–Gel ISFI systems. High viscosity of the gel matrix solutions affected the precipitation rate of the MS–Gel ISFI system and the encapsulation of the RM–MS into the gel matrix solutions. Increasing the viscosity of the gel matrix solution led to a decreased initial burst caused by a decreased drug diffusion rate both from the MS to the *in situ* gel and from the *in situ* gel to the dissolution medium. The amount of RM released from the MS–Gel ISFI in the initial 24 h increased in the rank order, S1 (SAIB/EtOH = 85/15, w/v) < S4 (SAIB/NMP = 75/25, w/v) < S2 (SAIB/EtOH = 80/20, w/v) < S3 (SAIB/EtOH = 75/25, w/v) < S6 (SAIB/NMP = 60/40, w/v), which is in good agreement with the order of viscosity of the gel matrix solutions (Figure S1). However, the system prepared with SAIB/NMP (70:30, w/v) had a higher initial burst but a higher viscosity compared to that prepared with SAIB/EtOH (75:25, w/v), which did not conform to the above rule. This could be attributed to a fast solvent exchange rate of NMP forming a porous implant structure, while a slow rate of EtOH resulted in a less porous structure during the phase inversion period (Thakur et al., 2014).

For the S1 formulation (RM–MS–Gel ISFI, SAIB/EtOH = 85/15, w/v), a first-order release model had the best fit with high correlation (R^2^>0.98). According to the results of the Korsmeyer–Peppas equation, the n value was between 0.45 and 0.89 (*n* = 0.71), indicating a non-Fickian drug release mechanism from the ISFI system. The release behavior of RM was controlled by a diffusion mechanism through water channels in the MS and gel matrix solution. In addition, matrix erosion of the PLGA polymer and degradation of the gel matrix contributed to this release behavior.

In conclusion, SAIB/EtOH (85/15, w/v), with a high viscosity, would be a suitable matrix solution for the preparation of RM–MS–Gel ISFI, according to the lowest burst release and the longest release time.

### Pharmacokinetic study

**Figure 4.**
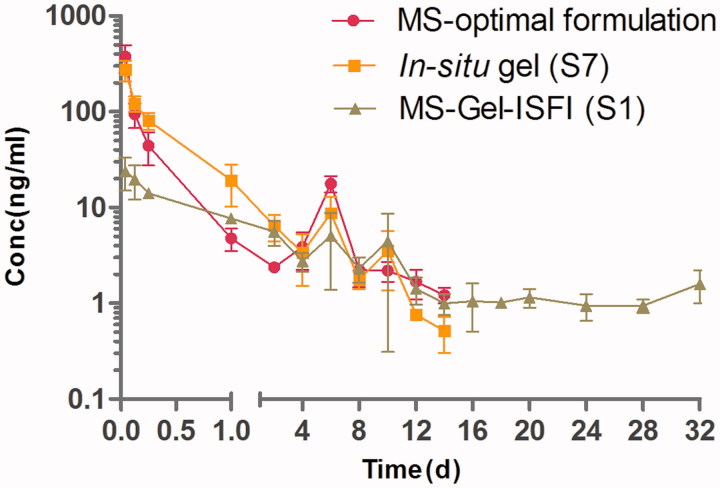
Mean plasma concentration-time curve of RM-microspheres with the optimal formulation, RM–*in situ* gel (gel matrix composed of 85% SAIB and 15% EtOH) with the drug loading of 30 mg/mL and RM–microsphere–Gel *in situ* forming implant (gel matrix composed of 85% SAIB and 15% EtOH) with the drug loading of 30 mg/mL after single-dose intramuscular injection of 5.6 mg/kg to rats (mean ± SD, *n* = 5).

Based on the results obtained from the plasma concentration–time profiles (Figure 4) and pharmacokinetic parameters (Table S4), it was found that the RM–MS–Gel ISFI system demonstrated a better pharmacokinetic character than the single RM–MS and RM–*in situ* gel systems did. The initial peak plasma drug concentration of RM–MS–Gel ISFI (26.69 ± 8.56 ng/mL) was significantly lower (*p* < .01) than that of both the RM–MS (374.91 ± 121.28 ng/mL) and RM–*in situ* gel (274.93 ± 66.04 ng/mL), 1 h after intramuscular administration. This indicates that RM–MS–Gel ISFI had a considerably slower initial drug release rate than RM–MS and RM–*in situ* gel did. In addition, the *C*_max_/*C*_ss_ of RM–MS–Gel ISFI (7.06 ± 1.16) was significantly lower (*p* < .01) than both the RM–MS (83.82 ± 27.06) and RM–*in situ* gel (79.75 ± 21.32) were, demonstrating an obviously reduced burst release (where *C*_max_ represents the maximum plasma concentration and *C*_ss_ represents the mean plasma concentration from days 2 to 32).

After the initial burst, the plasma concentration of the RM from both RM–MS and RM–*in situ* gel sharply declined two days after administration, and then dropped below the lowest limit of quantification for the analytical method (0.5 ng/mL) after 14 days. However, for the RM–MS–Gel ISFI, the RM demonstrated a sustained released in the range of 1.10-34.15 ng/mL, with a *C*_ss_ of 3.78 ± 0.64 ng/mL 2 days after administration; the plasma concentration of RM was still detectable after 32 days.

The elimination half-time (*T*_1/2_) of the RM from the RM–MS–Gel ISFI system (25.6 ± 26.3 days) was much longer compared to that of the single RM–MS (3.4 ± 2.2 days) and RM–*in situ* gel (3.5 ± 1.7 days) systems. In addition, no statistically significant differences in *C*_max_ and *T*_1/2_ were founded between RM–MS and RM–*in situ* gel. Dose-normalized AUC_0–∞_ values were relatively consistent, which demonstrates similar bioavailability of the three drug delivery systems.

These results highlight the benefit of using the sustained-release RM–MS–Gel ISFI system to provide a slow initial drug release and maintain a stable plasma concentration of RM for four weeks, which was in good agreement with the *in vitro* drug release performance.

### Pharmacodynamic study

**Figure 5.**
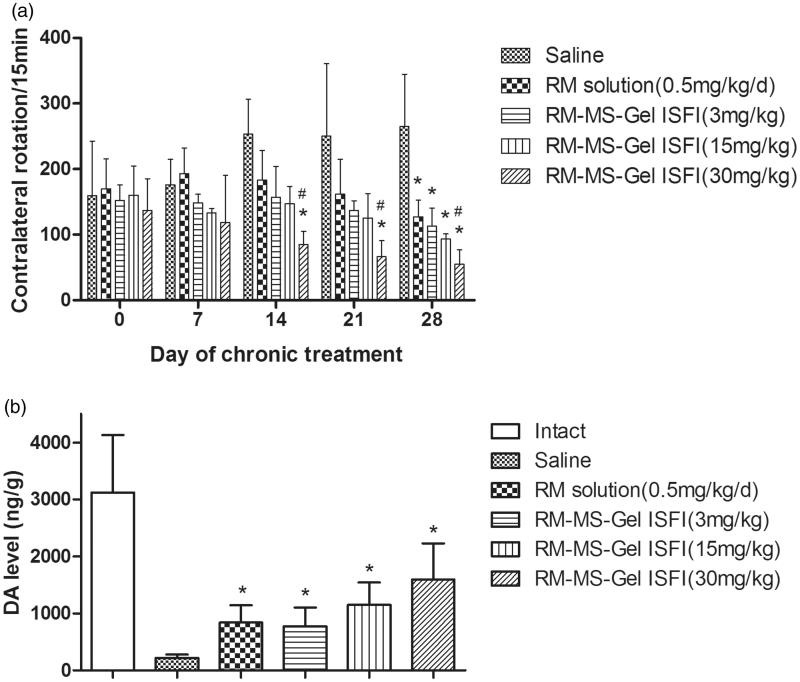
Effect of chronic treatment with RM–microsphere–Gel *in situ* forming implant on 6-hydroxydopamine-lesioned rats. (a) Rotational response to apomorphine expressed as number of contralateral rotations per 15 min in animals treated with saline and RM solution at 0.5 mg/kg/d (i.m.), and RM–microsphere–Gel *in situ* forming implant at a single-dose of 3, 15 and 30 mg/kg (i.m.). (b) Striatal DA level (ng/g wet weight of tissue) in intact side and lesioned side treated with saline, RM solution at 0.5 mg/kg/d, and RM–microsphere–Gel *in situ* forming implant at 3, 15 and 30 mg/kg after 28 days. Data shown are mean ± SD, (*n* = 5). *p < .05 for significant different from saline group and #*p* < .05 for significant different from RM solution group using one-way ANOVA followed by the Bonferroni test.

The therapeutic efficiency of RM–MS–Gel ISFI for PD treatment through intramuscular injection of different doses was evaluated in rats using lateral rotation tests and striatal DA level determination. Contralateral rotational behavior in the screening of the PD model showed no statistically significant difference among the groups on day 0. However, over a period of 28 days of chronic administration, animals in the saline group exhibited consistently increased rotational behavior, while the medicated groups showed a gradual decline in rotation after apomorphine induction (Figure 5(a)). Treatment of rats with RM solution (0.5 mg/kg/day) and RM–MS–Gel ISFI at different doses (3, 15, and 30 mg/kg) significantly attenuated these rotations compared to rotations in the saline group on day 28 (*p* < .05). These results demonstrate that the RM–MS–Gel ISFI system effectively counteracts progressive degeneration of the substantia nigra and striatum lesioned by 6-OHDA.

Additionally, the decrease in rotation of the high-dose RM–MS–Gel ISFI group reached statistical significance on days 14, 21, and 28 versus the saline group (*p* < .05), while the other groups showed no significant difference on days 14 and 21. There was also a significant difference between the high-dose RM–MS–Gel ISFI group and the RM solution group on days 14, 21, and 28. These results indicate that the high-dose RM–MS–Gel ISFI exhibits the best treatment efficacy.

Moreover, DA levels in the lesioned striatum were significantly higher in the RM-treated groups than in the saline group after 28 days (*p* < .05, Figure 5(b)). 6-OHDA injection caused over 90% depletion in the DA level of the lesioned side relative to that of the intact side, whereas the RM–MS–Gel ISFI system at different doses (3, 15, and 30 mg/kg) effectively restored the striatal DA level of PD rats to 25%, 37% and 52%, respectively.

## Conclusions

In the present study, an RM–MS–Gel ISFI system was prepared by dispersion of RM–MS into an *in situ* phase transition gel, and applied as a sustained-release depot of RM for the treatment of PD. *In vitro* drug release studies showed that, compared with that of the single RM–MS and RM–*in situ* gel systems, the RM–MS–Gel ISFI system reduced the initial drug burst and prolonged the release of RM for a period of 60 days. *In vivo* pharmacokinetics indicated that the *C*_max_ of RM–MS–Gel ISFI was significantly reduced compared with that of RM–MS and RM–*in situ* gel after intramuscular injection to rats. The results of the pharmacodynamic study demonstrated that animals treated with different doses of RM–MS–Gel ISFI (3, 15 and 30 mg/kg) effectively reduced turning behavior induced by apomorphine and effectively raised the DA level in the lesioned striatum. Finally, we suggest that MS-embedded *in situ* gel is superior for use as a biodegradable and injectable sustained drug delivery system with a low initial burst and long period of drug release for highly hydrophilic small-molecule drugs.

## Supplementary Material

IDRD_Sun_et_al_Supplemental_Content.docx

## References

[CIT0001] Afshin-Majd S, Bashiri K, Kiasalari Z, et al (2017). Acetyl-l-carnitine protects dopaminergic nigrostriatal pathway in 6-hydroxydopamine-induced model of Parkinson's disease in the rat. Biomed Pharmacother 89:1–9. 28199883 10.1016/j.biopha.2017.02.007

[CIT0002] Avachat AM, Kapure SS. (2014). Asenapine maleate in situ forming biodegradable implant: an approach to enhance bioavailability. Int J Pharm 477:64–72. 25305379 10.1016/j.ijpharm.2014.10.006

[CIT0003] Bege N, Renette T, Endres T, et al (2013). In situ forming nimodipine depot system based on microparticles for the treatment of posthemorrhagic cerebral vasospasm. Eur J Pharm Biopharm 84:99–105. 23298622 10.1016/j.ejpb.2012.12.016

[CIT0004] Bisht R, Jaiswal JK, Rupenthal ID. (2017). Nanoparticle-loaded biodegradable light-responsive in situ forming injectable implants for effective peptide delivery to the posterior segment of the eye. Med Hypotheses 103:5–9. 28571808 10.1016/j.mehy.2017.03.033

[CIT0005] Cavazzuti M. 2013. Design of experiments. 13–42.

[CIT0006] Collaborators IP. (2016a). Global, regional, and national incidence, prevalence, and years lived with disability for 310 diseases and injuries, 1990–2015: a systematic analysis for the Global Burden of disease study 2015. Lancet 388:1545–602. 27733282 10.1016/S0140-6736(16)31678-6 PMC5055577

[CIT0007] Collaborators MCOD. (2016b). Global, regional, and national life expectancy, all-cause mortality, and cause-specific mortality for 249 causes of death, 1980–2015: a systematic analysis for the Global Burden of disease study 2015. Lancet 388:1459–544. 27733281 10.1016/S0140-6736(16)31012-1 PMC5388903

[CIT0008] Connolly BS, Lang AE. (2014). Pharmacological treatment of Parkinson disease: a review. JAMA 311:1670 24756517 10.1001/jama.2014.3654

[CIT0009] Dashtipour K. (2007). Comprehensive review of rasagiline, a second-generation monoamine oxidase inhibitor, for the treatment of Parkinson's disease. Clin Therapeut 29:1825. 10.1016/j.clinthera.2007.09.021 18035186

[CIT0010] David B, Fong JW, Thomas K, et al 1997. Sustained release formulations of water soluble peptides.

[CIT0011] Ellis JM, Fell MJ. (2017). Current approaches to the treatment of Parkinson’s disease. Bioorg Med Chem Lett 27:4247–55. 28869077 10.1016/j.bmcl.2017.07.075

[CIT0012] Fernandez M, Barcia E, Fernandez-Carballido A, et al (2012). Controlled release of rasagiline mesylate promotes neuroprotection in a rotenone-induced advanced model of Parkinson's disease. Int J Pharm 438:266–78. 22985602 10.1016/j.ijpharm.2012.09.024

[CIT0013] Fernandez M, Barcia E, Negro S. (2009). Development and validation of a reverse phase liquid chromatography method for the quantification of rasagiline mesylate in biodegradable PLGA microspheres. J Pharm Biomed Anal 49:1185–91. 19356876 10.1016/j.jpba.2009.02.031

[CIT0014] Fernandez M, Negro S, Slowing K, et al (2011). An effective novel delivery strategy of rasagiline for Parkinson's disease. Int J Pharm 419:271–80. 21807080 10.1016/j.ijpharm.2011.07.029

[CIT0015] Garbayo E, Ansorena E, Blanco-Prieto MJ. (2013). Drug development in Parkinson's disease: from emerging molecules to innovative drug delivery systems. Maturitas 76:272. 23827471 10.1016/j.maturitas.2013.05.019

[CIT0016] Juvekar S, Kathpalia H. (2017). Solvent removal precipitation based in situ forming implant for controlled drug delivery in periodontitis. J Control Release 251:75–81. 28242417 10.1016/j.jconrel.2017.02.022

[CIT0017] Karfeld-Sulzer LS, Ghayor C, Siegenthaler B, et al (2015). N-methyl pyrrolidone/bone morphogenetic protein-2 double delivery with in situ forming implants. J Control Release 203:181–8. 25697800 10.1016/j.jconrel.2015.02.019

[CIT0018] Lin X, Xu Y, Tang X, et al (2015). A uniform ultra-small microsphere/SAIB hybrid depot with low burst release for long-term continuous drug release. Pharm Res 32:3708–21. 26077999 10.1007/s11095-015-1731-1

[CIT0019] Ma J, Chen X, Duan X, et al (2008). Validated LC–MS/MS method for quantitative determination of rasagiline in human plasma and its application to a pharmacokinetic study. J Chromatogr B 873:203–8. 10.1016/j.jchromb.2008.08.024 18799367

[CIT0020] Marsili L, Marconi R, Colosimo C. (2017). Treatment strategies in early Parkinson's disease. Int Rev Neurobiol 132:345–60. 28554414 10.1016/bs.irn.2017.01.002

[CIT0021] Ng SM, Choi JY, Han HS, et al (2010). Novel microencapsulation of potential drugs with low molecular weight and high hydrophilicity: hydrogen peroxide as a candidate compound. Int J Pharm 384:120–7. 19819316 10.1016/j.ijpharm.2009.10.005

[CIT0022] Paillard-Giteau A, Tran VT, Thomas O, et al (2010). Effect of various additives and polymers on lysozyme release from PLGA microspheres prepared by an s/o/w emulsion technique. Eur J Pharm Biopharm 75:128–36. 20226857 10.1016/j.ejpb.2010.03.005

[CIT0023] Ravi PR, Aditya N, Patil S, Cherian L. (2015). Nasal in-situ gels for delivery of rasagiline mesylate: improvement in bioavailability and brain localization. Drug Deliv 22:903–10. 24286183 10.3109/10717544.2013.860501

[CIT0024] Thakur RRS, Mcmillan HL, Jones DS. (2014). Solvent induced phase inversion-based in situ forming controlled release drug delivery implants. J Control Release 176:8–23. 24374003 10.1016/j.jconrel.2013.12.020

[CIT0025] Wang L, Wang A, Zhao X, et al (2012). Design of a long-term antipsychotic in situ forming implant and its release control method and mechanism. Int J Pharm 427:284–92. 22387369 10.1016/j.ijpharm.2012.02.015

[CIT0026] Wang P, Zhuo X, Chu W, Tang X. (2017). Exenatide-loaded microsphere/thermosensitive hydrogel long-acting delivery system with high drug bioactivity. Int J Pharm 528:62–75. 28579543 10.1016/j.ijpharm.2017.05.069

[CIT0027] Wang T, Yang L, Hua J, et al (2016). Simultaneous bioanalysis of rasagiline and its major metabolites in human plasma by LC–MS/MS: application to a clinical pharmacokinetic study. J Pharm Biomed Anal 125:280–5. 27060436 10.1016/j.jpba.2016.04.003

[CIT0028] Weinreb O, Badinter F, Amit T, et al (2015). Effect of long-term treatment with rasagiline on cognitive deficits and related molecular cascades in aged mice. Neurobiol Aging 36:2628–36. 26142126 10.1016/j.neurobiolaging.2015.05.009

[CIT0029] Yoram S, Nurit L, Itschak L, Tomer M. 2012. Extended release formulations of rasagiline and uses thereof US.

