# Supplementary material for: Preparation and evaluation of injectable Rasagiline mesylate dual-controlled drug delivery system for the treatment of Parkinson’s disease
Source: Drug Deliv. 2017 Dec 23;25(1):143–52. doi: 10.1080/10717544.2017.1419514 (PMC6058670; doi:10.1080/10717544.2017.1419514)
Supplement: IDRD_Sun_et_al_Supplemental_Content.docx [file IDRD_A_1419514_SM2007.docx]

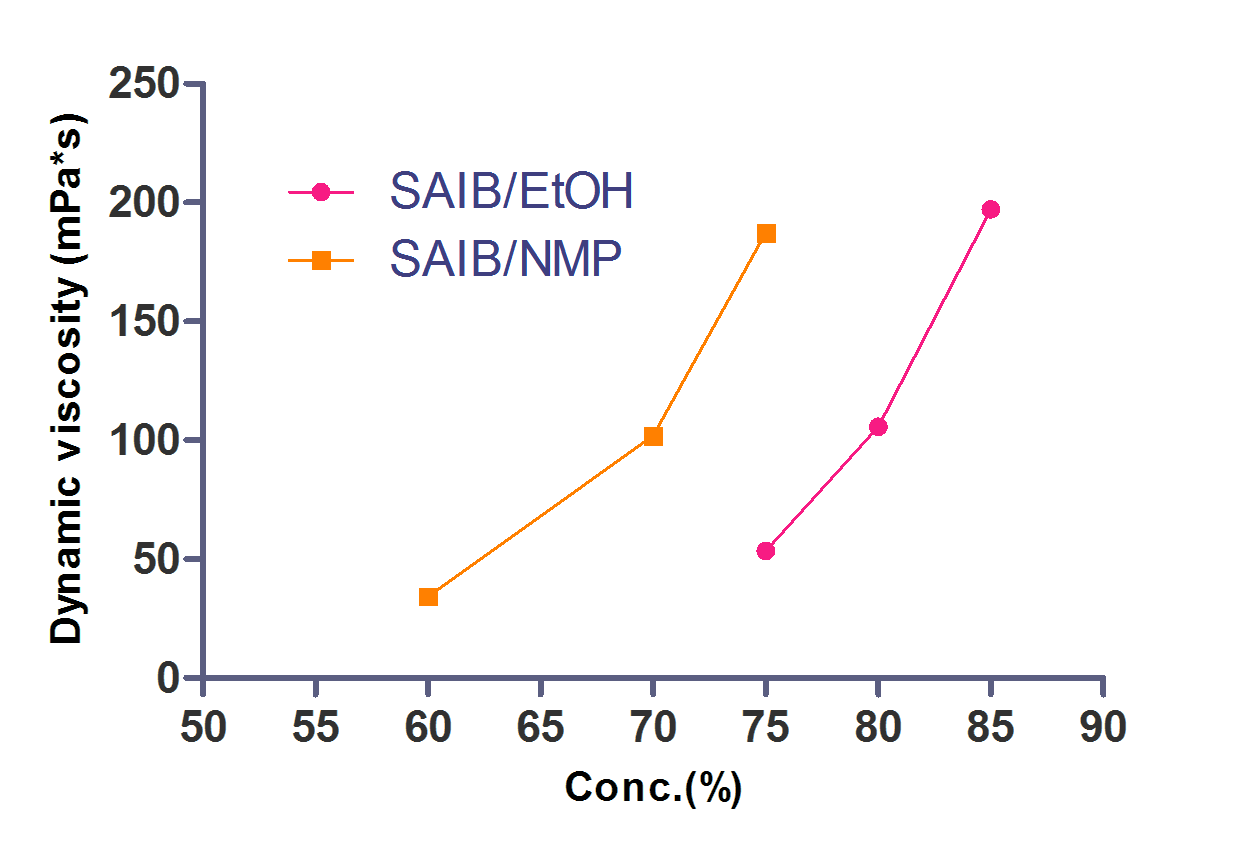


**Figure S1.** Relative viscosities of gel matrix solutions in different concentration of EtOH and NMP solvents.

**Table S1.** Variables and levels in Box-Behnken design

| Independent variables | Levels | | | Dependent variables | Constraints |
| --- | --- | --- | --- | --- | --- |
|  | -1 | 0 | 1 |  |  |
| X_1_= drug: polymer ratio | 1:2.3 | 1:1.8 | 1:1.5 | Y_1_=drug loading content (%) | Maximize |
| X_2_=organic phase: aqueous phase ratio | 1:1.5 | 7.5:1 | 9:1 | Y_2_=encapsulation efficiency (%) | Maximize |
| X_3_=stirring speed (rpm) | 200 | 250 | 300 | Y_3_=particle size (μm) | Minimum |

**Table S2.** The properties of RM-MS of different formulations

| Formulations | | | | Theoretical DL (%) | Actual DL (%) | EE (%) | Particle size (μm) | Span |
| --- | --- | --- | --- | --- | --- | --- | --- | --- |
| A | single emulsion | O/W |  | 9.13 | 1.41±0.05 | 15.48±0.49 | 28.34±0.70 | 1.219 |
| B | double emulsion | W/O/W |  | 3.29 | 2.57±0.07 | 78.22±2.13 | 55.22±0.74 | 1.271 |
| C | double emulsion | S/O/W |  | 3.30 | 1.01±0.09 | 30.61±2.59 | 33.45±2.68 | 1.370 |
| D | traditional phase separation | O/O |  | 9.35 | 2.86±0.14 | 30.59±1.50 | 46.62±2.66 | 1.295 |
| E | emulsion-phase separation | W/O/O | E1 | 9.10 | 7.20±0.02 | 79.12±0.22 | 45.38±0.72 | 1.091 |
|  |  |  | E2 | 20.00 | 17.34±0.11 | 86.55±0.55 | 50.04±0.84 | 1.001 |
|  |  |  | E3 | 30.00 | 26.42±0.81 | 88.07±2.69 | 62.56±1.18 | 1.330 |

**Table S3.** Composition and observed responses in Box-Behnken design

| Std | Run | Independent Variables | | | Dependent Variables | | |
| --- | --- | --- | --- | --- | --- | --- | --- |
|  |  | X1 | X2 | X3 | Y1 | Y2 | Y3 |
|  |  | Drug:polymer ratio | Organic: aqueous phase ratio | Stirring speed (rpm) | DL (%) | EE (%) | Particle size (μm) |
| 2 | 1 | 1:1.5 | 6:1 | 250 | 8.23 | 20.59 | 98.557 |
| 17 | 2 | 1:1.8 | 7.5:1 | 250 | 29.50 | 84.16 | 62.086 |
| 13 | 3 | 1:1.8 | 7.5:1 | 250 | 30.10 | 85.88 | 63.870 |
| 5 | 4 | 1:2.3 | 7.5:1 | 200 | 24.11 | 80.40 | 92.799 |
| 4 | 5 | 1:1.5 | 9:1 | 250 | 12.03 | 30.08 | 105.007 |
| 12 | 6 | 1:1.8 | 9:1 | 300 | 26.62 | 75.27 | 92.086 |
| 7 | 7 | 1:2.3 | 7.5:1 | 300 | 25.58 | 85.35 | 79.189 |
| 9 | 8 | 1:1.8 | 6:1 | 200 | 22.97 | 65.50 | 103.991 |
| 1 | 9 | 1:2.3 | 6:1 | 250 | 24.89 | 82.99 | 90.614 |
| 14 | 10 | 1:1.8 | 7.5:1 | 250 | 28.98 | 82.56 | 72.891 |
| 10 | 11 | 1:1.8 | 9:1 | 200 | 29.33 | 83.69 | 97.876 |
| 3 | 12 | 1:2.3 | 9:1 | 250 | 23.88 | 79.62 | 71.417 |
| 16 | 13 | 1:1.8 | 7.5:1 | 250 | 29.51 | 84.26 | 75.263 |
| 6 | 14 | 1:1.5 | 7.5:1 | 200 | 11.96 | 29.92 | 118.197 |
| 15 | 15 | 1:1.8 | 7.5:1 | 250 | 28.78 | 82.12 | 72.891 |
| 8 | 16 | 1:1.5 | 7.5:1 | 300 | 13.66 | 34.15 | 73.627 |
| 11 | 17 | 1:1.8 | 6:1 | 300 | 27.32 | 77.79 | 82.207 |

**Table S4.** Pharmacokinetic parameters of RM after intramuscular administration of RM-MS (optimal formulation), RM-*in-situ* gel (S7) and RM-MS-Gel ISFI (S1) to rats at dose of 5.6mg/kg (mean±SD; n=5)

| Parameters | RM-MS | RM-*in-situ* gel | RM-MS-Gel ISFI |
| --- | --- | --- | --- |
| T_max_ (h) | 1±0 | 1±0 | 1.67±1.16 |
| C_max_ (ng/mL) | 374.91±121.28 | 274.93±66.04 | 26.69±8.56^**##^ |
| C_ss_ (ng/mL) | 4.47±0.25 | 3.59±1.26 | 3.78±0.64 |
| C_max_/C_ss_ | 83.82±27.06 | 79.75±21.32 | 7.06±1.16^**##^ |
| AUC_0-t_ (ng·h/mL) | 2806.46±464.20 | 3084.54±747.35 | 1852.50±476.61 |
| AUC_0-∞_ (ng·h/mL) | 2946.08±553.37 | 3154.10±701.03 | 3084.01±1092.48 |
| MRT_0-∞_(h) | 102.02±10.56 | 67.62±17.53 | 857.88±699.97 |
| T_1/2_(h) | 80.98±53.79 | 84.03±41.59 | 614.736±330.54 |

^**^Indicates the values are significant different from RM-MS at p˂0.01.

^##^Indicates the values are significant different from RM-*in-situ* gel at p˂0.01.
